# Path-oriented test cases generation based adaptive genetic algorithm

**DOI:** 10.1371/journal.pone.0187471

**Published:** 2017-11-14

**Authors:** Xiaoan Bao, Zijian Xiong, Na Zhang, Junyan Qian, Biao Wu, Wei Zhang

**Affiliations:** 1 Department of Information Technology, Zhejiang Sci-Tech University, Hangzhou, China; 2 Guangxi Key Laboratory of Trusted Software, Guilin University of Electronic Technology, Guilin, China; Universita degli Studi di Catania, ITALY

## Abstract

The automatic generation of test cases oriented paths in an effective manner is a challenging problem for structural testing of software. The use of search-based optimization methods, such as genetic algorithms (GAs), has been proposed to handle this problem. This paper proposes an improved adaptive genetic algorithm (IAGA) for test cases generation by maintaining population diversity. It uses adaptive crossover rate and mutation rate in dynamic adjustment according to the differences between individual similarity and fitness values, which enhances the exploitation of searching global optimum. This novel approach is experimented and tested on a benchmark and six industrial programs. The experimental results confirm that the proposed method is efficient in generating test cases for path coverage.

## Introduction

Automatic software testing is among the most studied topics in the field of search-based software engineering (SBSE) [1–3]. One critical task in software testing is to generate test data to satisfy given adequacy criteria, among which white box testing is one of the most widely known. Given a coverage criterion, the challenge of generating test cases is to search for a set of data that lead to the highest coverage when given as input to the software under test. Various techniques for generating test cases have been developed in recent years, and the use of heuristic search techniques has been a burgeoning interest for many researchers. In the generation of test cases using heuristic search, feedback information concerning the tested application is used to determine whether the test data meet the testing requirements. The feedback mechanism gradually adjusts test data input until test requirements are met. This kind of method was proposed in [4], and has led to a considerable amount of subsequent research [5–8]. Fu B proposed a kind of software test data automated generation method based on simulated annealing genetic algorithm [27].An optimized technique for test case generation using tabu search and data clustering was proposed in [28].

Among these heuristics, genetic algorithms are the most widely used [8]. This paper is focused on structural testing at the unit level using genetic algorithm. In 1992, the GA was first applied to the automatic generation of test data in structural testing [9]. In 2001, Wegener [10] put forward a kind of automatic test cases generation technique based on genetic algorithm, which enhance test cases on the basis of certain control flow coverage for achieving a higher coverage. In 2008, Alba analyzed the application of parallel and sequential evolutionary algorithms to the automatic test data generation problem [11]. In 2009, Awedikian [12] presented a kind of extended branch distance calculation method by using the genetic algorithm framework, with dependency relationships among variables to guide test cases generation. In 2012, Maragathavalli presented an evolutionary method for test data generation with multiple paths coverage for instrumented programs by [13]. In 2013, Pachauri [14] provided an approach using branch ordering, memory and elitism for branch testing using genetic algorithm to improve test data generation performance. In 2014, R. Girgis [15] put forward a structural-oriented technique that uses a genetic algorithm (GA) for automatic generation of a set of test paths that cover the all-uses criterion, and the experiments show better test data generation performance compared to the random method. Mei Jia and Wang Shengyuan discuss a method that can automatically generate test cases for selected paths using an improved genetic algorithm and a comparative experiment results prove a great improvement in optimization efficiency [16].

However, in recent years, it has been acknowledged that the performance of GAs depend on the numerous parameters, such as crossover rate *P*_*c*_ and mutation rate *P*_*m*_ [17–19]. Algorithm parameters determine how the search space is explored, and poor algorithm parameterization hinders the discovery of solutions with high quality due to the influence of parameter values on algorithm performance [20–21]. Unfortunately, the configuration of the algorithm is usually the responsibility of the practitioner, who often is not an expert in search-based algorithm. Further still, the population of GAs may fall into a local optimum due to the sharp decline in diversity in the later phase [22], which inevitably hinders automatic test case generation using the GAs. To address the above issues, in this study, we introduce an adaptive evolutionary algorithm, which adjusts crossover rate and mutation rate in a dynamical way during the optimization process by maintaining population diversity.

## Materials and methods

### Test case generation

#### The framework of test case generation based on GAs

**Fig 1:**
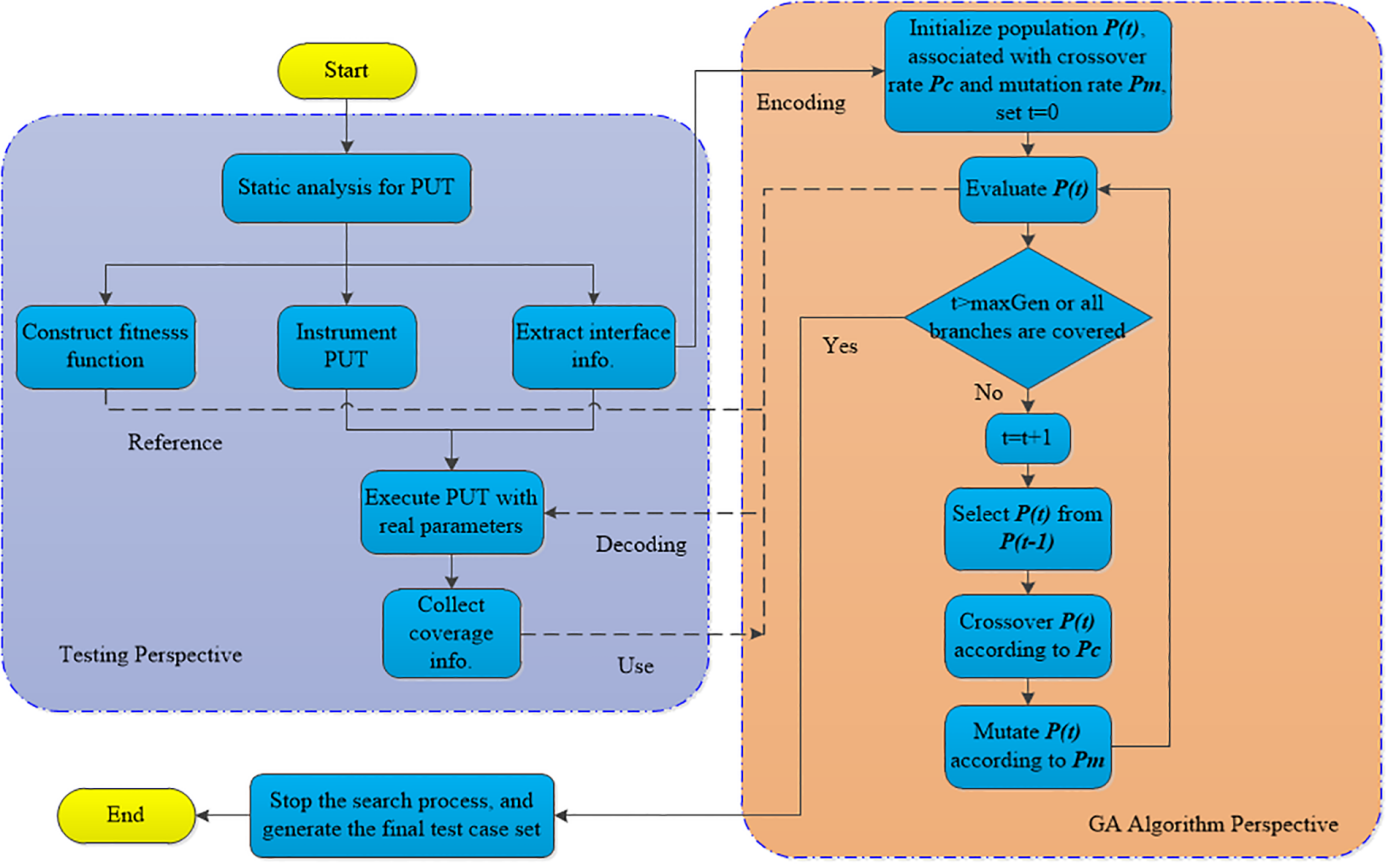
The automatic generation of test case model based on genetic algorithm.

Genetic algorithms (GAs) are proposed by Holland in his book Adaptation in Natural and Artificial Systems in 1975 on the concept of survival of fittest. They are the most famous metaheuristic used in the field of search-based software engineering [8]. The core problem in test case generation method based on the genetic algorithm is ensuring the collaborative operation of the GA and the testing process. The algorithm can be decomposed into two parts (Fig 1): a test perspective and a GA search perspective. The basic process of the algorithm is as follows: The following tasks should be performed first based on the testing program to be measured in a static analysis: a) the extraction of interface information, b) the formation of instrumentation to the corresponding program’s structural elements given the test adequacy criteria *C*, and c) the structuring of fitness function according to the adequacy criteria *C*. The input parameters of the program are then coded into individual gene vectors. At the same time, we initialize population *P*(*t*) associated with crossover rate *P*_*c*_ and mutation rate *P*_*m*_, and set *t* = 0, where *P*(*t*) denotes the *t*th generation within population *P*. Following this, the decoded individuals are entered as input parameters to drive the testing program and collect the corresponding coverage information. The fitness value for each individual can then be calculated to obtain *P*(*t*) using the input parameters associated with the coverage information. Finally, *P*(*t*) is updated with some genetic operators (selection, crossover and mutation) until the termination condition is satisfied.

In this algorithm, the terminating conditions are set for one of two situations as follows in general: 1) evolution generation reaches *maxGen*, or 2) the generated test case set realizes the complete coverage of the target covering all elements (statement, branch or path).

From a testing perspective, the process involves two core components: a static parser and a test driver. 1) The static parser is mainly responsible for lexical and syntactical analysis of program code for the design of the instrument and the adaptive function. 2) The test driver is mainly used to enter the parameters into the application being tested and collect the coverage information of the structural elements to calculate the corresponding fitness.

From the perspective of the GA, it uses feedback according to the fitness value to guide the update of the population. It focuses on genetic operators, such as selection, crossover and mutation, to decode the formal parameter into actual parameters. We can hence learn the superiority of the given solution vectors through the test driver.

#### Fitness calculation

The path-oriented method is widely used in structural testing because it is cost effective [23]. Path-oriented methods require execution of certain paths in the control-flow graph. Our test data generator breaks down the global objective (to cover all the branches) into several partial objectives consisting of dealing with one target path containing several branches of the program. The challenge of test cases generation using GAs can be converted to an optimization problem of generating test data covering the target path, and the design of fitness function is the key to solve it. For covering individual branches, the fitness function is a function Fitness(x,t)→R, that takes a target path *t* and individual input ***x***, where ***x*** = (*x*_1_, *x*_2_, ⋯ *x*_*len*_) is a vector of the input variables of the function under test, and the domain $D_{x_{n}}$ of the input variables *x*_*n*_ is a set of all values that *x*_*n*_ can hold, 1 ≤ n ≤ *len*; *len* = |***x***|. In fact, the calculation of the fitness function involves two components: the so-called *approach level* and *branch distance* [10].

**Fig 2:**
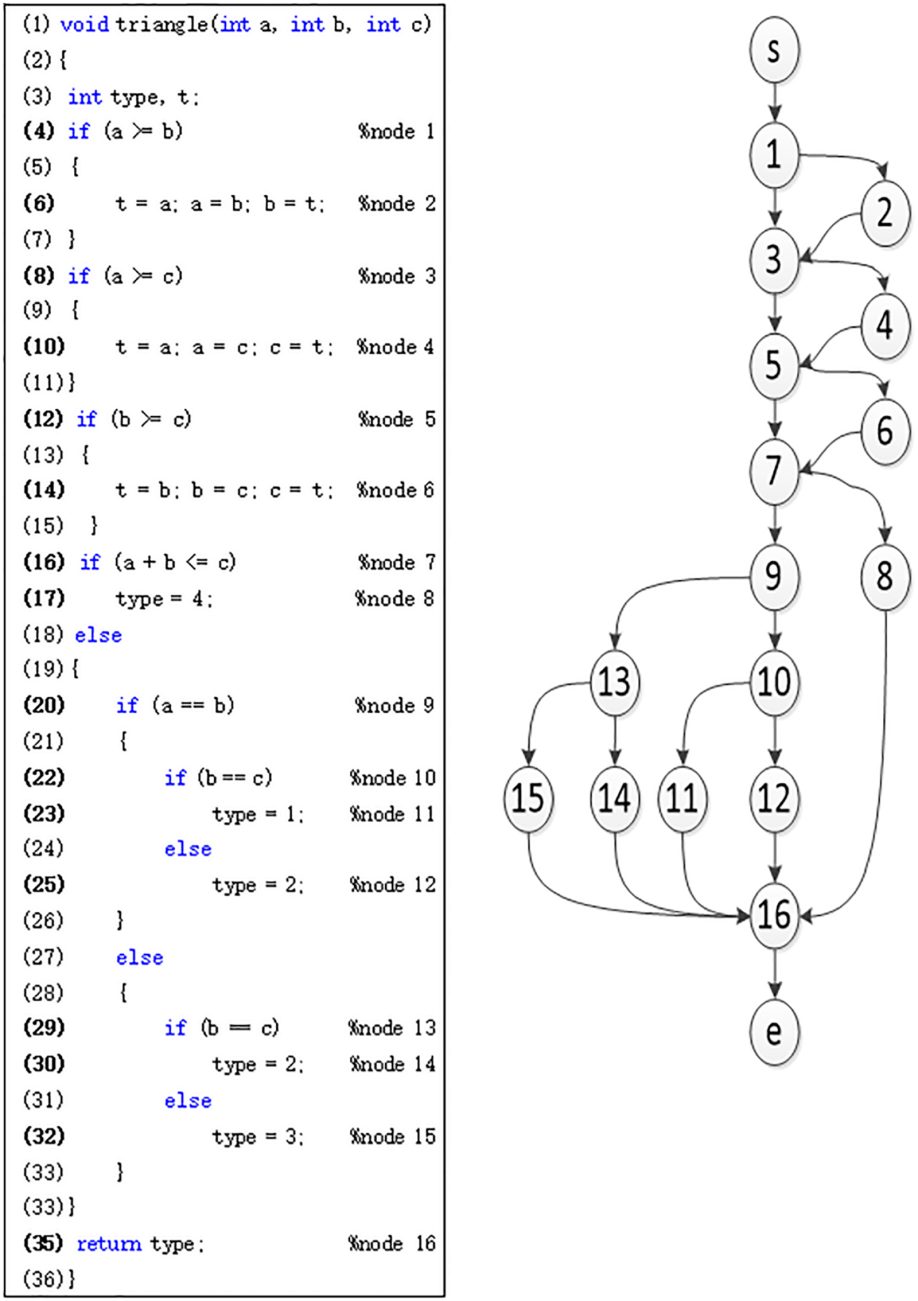
Triangle type program and its corresponding control flow graph.

The approach level assesses the path taken by the input with respect to the target branch by calculating the target’s control dependencies that were not executed by the path. Fig 2 shows code and the control flow graph of a program that classify triangles in four types. Suppose the *i*th individual ***x***_***i***_ through the path P(***x***_***i***_), and the target path is P(target). The approach level for fitness calculation can be calculated by formula (1),

$$approach\;level(x_{i})\;=\;\frac{\alpha (x_{i})}{\left|P(target)\right|}$$ (1)

where *α*(***x***_***i***_) is the number of untraversed nodes of the path P(***x***_***i***_) with respect to the target path P(target), and |P(target)| is the number of nodes for target structural path when ***x***_***i***_ as a input to execute the program under test. For example, suppose P(***x***_***i***_) = "s → 1,2,3,4,5,7,9,10,12,16 → e", and P(target) = "s → 1,2,3,4,5,6,8,16 → e", then we can calculate the approach level (***x***_***i***_) by $"approach\;level(x_{i})\;=\;\frac{\alpha (x_{i})}{\left|P(target)\right|}\;=\;\frac{2}{9}"$.

For the component of the branch distance for fitness calculation, in this paper, we draw lessons from Korel and Tracey’s work [5–6]. When execution of a test case diverges from the target branch, the branch distance expresses how close an input came to satisfying the condition of the predicate at which control flow for the test case went ‘wrong’; that is, how close the input was to descending to the nest approach level. Some typical branch distance functions are shown in Table 1, where *K* represents a positive number such that the objective function always returns a value no smaller than zero when the result of predicate calculation is false. Since the maximum branch distance is generally not known, the standard approach to normalization cannot be applied [24]; instead the following formula is used:

$$normalize(branch\;distance)\;=\;1-1.001^{-branch\;distance}$$ (2)

**Table 1:** Branch distance functions for several kinds of branch predicates.

No.	Branch Predicate	Branch distance function *f*(*bch*_*i*_)
1	*a = b*	If |*a* − *b*| = 0 *then* 0 *else* |*a* − *b*| + *K*
2	*a*! = *b*	If |*a* − *b*| ≠ 0 *then* 0 *else K*
3	*a < b*	If *a* − *b* < 0 *then* 0 *else* (*a* − *b*) + *K*
4	*a* ≤ *b*	If *a* − *b* ≤ 0 *then* 0 *else* (*a* − *b*) + *K*
5	*a > b*	If *b* − *a* < 0 *then* 0 *else* (*b* − *a*) + *K*
6	*a* ≥ *b*	If *b* − *a* ≤ 0 *then* 0 *else* (*b* − *a*) + *K*
7	*a* ∩ *b*	*f*(*a*) *+ f*(*b*)
8	*a* ∪ *b*	*min* (*f*(*a*), *f*(*b*))
9	! *a*	*negation is propagated over a*
10	*boolean*	If *true then* 0 *else K*

The fitness function of the entire testing program can then be calculated by approach level and normalizing the branch distance according to Table 1:

$$Fitness\left(x,t\right)=\frac{1}{\varepsilon +approach\;level\left(x,\;\text{t}\right)+\sum_{i=1}^{s}w_{i}\cdot normalize\left(f\left(bch_{i}\right)\right)}$$ (3)

The *Fitness*(***x***, *t*) value is assigned to each chromosome (individual input) ***x*** for the target path t. Of these, *s* is the number of branches of the target path, *w*_*i*_ is the weight for each branch and ε is a minimum constant set to 0.01 in our experiment. Usually, the difficulty in reaching each individual differs among branches; thus, the weights should be set differently for them.

In general, the greater the nesting degree (*nd*) of a branch, the more difficult it is to arrive at the branch; hence, the weight of the branch should be increased [25]. We can obtain the nesting degree of *bch*_*i*_ (1 ≤ *i* ≤ *s*) by static program analysis. Here, we assume that the maximum nesting degree is *nd*_*max*_ and the minimum is *nd*_*min*_; then, the nesting weight of *bch*_*i*_ can be calculated by (4):

$$w_{i}\;=\;\frac{nd_{i}-nd_{min}+1}{nd_{max}-nd_{min}+1}$$ (4)

### Adjusting parameters of GAs based on population diversity

There are two different ways for configuring parameter values in the field of software engineering: by tuning their values before the optimization process, or by dynamically adjusting parameter assignments during the run. The later is referred to as parameter control, and is more effective than parameter tuning because adaptive parameter control does not use a predefined schedule and does not extend the solution size [19]. In this section, we first introduce a population diversity metric method, and use it to design adaptive genetic operators by adjusting parameters in a dynamic way.

#### Population diversity metric method

Premature convergence has been an important problem for the classic genetic algorithm. Studies have shown that there is a close relationship between premature convergence and lack of population diversity. The reasonable and effective depiction of population diversity is an important problem for evolutionary algorithms. The Hamming distance is a common method to define it, but it does not take the information of individual fitness into consideration [29]. This paper provides a population diversity metric that considers both the effects of them.

**Definition 1:** *Similarity among individuals*, *SAI*: It is used for the quantitative description of the similarity between genes, which can be depicted by the Hamming distance [30]. The Hamming distance *d*_*ij*_ between individual *i* and *j* can be defined as follows:

$$d_{ij}\;=\;\|x_{i}-x_{j}\|\;=\;\sqrt{\sum_{k\;=\;1}^{N}{\left(x_{ik}-x_{jk}\right)}^{2}}(i,j\;=\;1,2\cdots n)$$ (5)

In this formula, *x*_*i*_ denotes the *i*th input variable and *x*_*ik*_ the *k*th symbol (‘0’ or ‘1’) of the binary string in the *i*th input variable, *N* is the length of the binary string, and *n* is the number of individuals. The Hamming distance of the *i*th individual within the population can be calculated by (6):

$$d_{i}\;=\;\sum_{j\;=\;1}^{n}d_{ij}$$ (6)

Then the Hamming distance of population is:

$$D_{Hamming}\;=\;\sum_{i\;=\;1}^{n}\sum_{j\;=\;1}^{n}d_{i}$$ (7)

**Definition 2:** *Degree of population diversity*, *DPD*: It describes the diversity and universality of the individuals genetic in a population, which measured by the Hamming distance between the individuals and the variance of fitness value. We assume that population P containing *M*_*k*_ individuals is denoted by $K\;=\;\{X_{1},X_{2},\cdots X_{M_{k}}\}$. Each individual *X*_*i*_ corresponds to a special fitness value, and the average fitness of the *k*th population can be calculated by (8).

$$f_{avg}^{k}\;=\;\frac{\sum_{i\;=\;1}^{M_{k}}f_{i}}{M_{k}}$$ (8)

*DPD* is defined as in (9):

$$DPD≝\rho ∙D_{Hamming}+(1-\rho )\frac{1}{M_{k}}\sum_{i\;=\;1}^{M_{k}}{\left(f_{i}^{k}-f_{avg}^{k}\right)}^{2}$$ (9)

The ρ(0 ≤ ρ ≤ 1) is the weighting parameter.

#### Design of genetic operators

Genetic operators are essential to obtain the next generation of a given population and crucial for evolutionary iteration. The classical GA with fixed values of crossover rate and mutation rate encounter the search stagnation phenomenon in their later phases due to a lack of diversity in the population. We adopt the method of dynamic adjustment of genetic operators (selection operator, crossover operator, mutation operator) to improve the intelligence and effectiveness of the algorithm.

Selection operator is used to determine the chromosomes to be used as parents in the creation of the offspring that populate the subsequent generation. Methods of choice by probability mimic the survival of the fittest, and the roulette wheel method is one of the widely used in GAs. However, this fitness-proportionate selective mechanism has difficulties in maintaining a constant selective pressure through the search, which is the probability of the best individual being selected, compared to the average probability of selection of all individuals. In the first few generations of the search, fitness variance is usually high, since the most highly-fit individuals will be granted the greatest opportunities to become parents because of the corresponding high selective pressure. This can lead to premature convergence. Besides that, in later generations of the search, when fitness values among individuals are similar and the fitness variance is correspondingly low, selective pressure is also low. This can lead to stagnation of the search.

Linear ranking of individuals is a method which proposes to circumvent the above problem. Individuals are ranked according to fitness and assigned an intermediate fitness value based on their rank, rather than through the direct use of fitness value. A linear ranking mechanism with value *Z*, where 1 < *Z* ≤ 2, allocates a selective value of *Z* to the best individual, a value of 1.0 to the median individual, and the worst individual receiving a value of 2 − *Z*. Parents are then chosen two at a time for crossover using stochastic universal sampling, such that each individual has a probability of being selected proportionate to its intermediate linearly-ranked fitness value [10]. This paper used the linear rank method as selection operator in our experiments which assign *Z* a value of 1.7.

The crossover operation selects the point at which material from two parents combines to create two new offspring. In one-point crossover, a single crossover point is chosen at random. A recombination of two individuals <0, 127, 0> and <127, 0, 127> in the range [0,127], ‘000000011111110000000’ and ‘111111100000001111111’ in encoded form, with a single-point crossover chosen to take place at locus 9, would take place as follows:

This produces two offspring, <0, 96, 127> and <127, 31, 0>.

The operation is regulated by the probability of the crossover parameter *P*_*c*_. The crossover rate is the guarantee of population diversity. If the crossover rate is set too large, good individual genes with high values of fitness can be destroyed easily; if it is set too small, it causes slow search in producing a new individual. We assume that the parents are *x*_*i*_ and *x*_*j*_ due to the selection operator, and the crossover rate, *P*_*c*_ can be given as

$$P_{c}=\{\begin{array}{ll} P_{c0}\left(1-normalize\left(DPD\right)\right) & ,f^{\prime }\geq f_{avg}\\ 1 & ,f^{\prime }<f_{avg} \end{array}$$ (10)

where 0 < *P*_*c*0_ ≤ 1.0 and *f*′ is the larger of the fitness values of the individual parents. To avoid *P*_*c*_ > 1 when *f*' ≥ *f*_*avg*_, the given *Degree of population diversity* (*DPD*) should be normalized. There are many methods to normalize *DPD*, and one is: $normalize(DPD)\;=\;\frac{DPD}{DPD+\beta }\;(\beta \;is\;a\;constant\;and\;\beta >0)$, which is used in our experiment. In fact, although different methods of normalization maybe a optimizing point, it is not discussed in this paper. The formula of *P*_*c*_ reflects the fact that when the *DPD* is low, the crossover rate should be increased accordingly to improve population diversity when *f*′ is no less than *f*_*avg*_ so as to prevent premature convergence. When *f*′ is less than *f*_*avg*_, *P*_*c*_ is set 1.0 to avoid the over-optimization of the parameters to the solution.

The main purpose of the mutation operator is to improve the local search capability of the GAs to maintain the diversity of the population. It is usually achieved by flipping bits of the binary strings at some probability rate *P*_*m*_. On the one hand, it is difficult to produce new individuals and the population diversity cannot be guaranteed if the mutation rate is set too small. On the other hand, it may cause considerable damage to genes, such that the GA degrades into a random search algorithm. We use an adaptive mutation probability according to *Degree of population diversity* (*DPD*). We assume that the gene *x*_*i*_ is associated with the fitness value *f*_*i*_; its mutation rate *P*_*m*_ can be given as

$$P_{m}=\{\begin{array}{ll} P_{m0}\left(1-normalize\left(DPD\right)\right) & ,f_{i}\geq f_{avg}\\ P_{m0} & ,f_{i}<f_{avg} \end{array}$$ (11)

where 0 < *P*_*m*0_ ≤ 1.0. Generally, *P*_*m*0_ is set to a small value, and the mutation rate can be changed adaptively to maintain population diversity according to the individual diversity level while *f*_*i*_ is no less than *f*_*avg*_.

#### IAGA implementation of the algorithm

For convenience of description, we call the improved method for generating test cases based on adaptive genetic algorithm IAGA. The implementation of the pseudo code of IAGA is given as follows in Algorithm 1.

**Algorithm 1:** Test cases generation based on improved adaptive genetic algorithm (IAGA).

1: **Input** Program: instrumented version of a program to be tested 2: CDG: control-dependence graph for PUT (Program Under Test) 3: Partial objective: one specific target path of TestReq 4: *f*- fitness function; *M_k_*- population size; *SC*- stop criterion 5: *ρ*- the weighting parameter for computing *DPD*(*Degree of Population Diversity*) 6: *z*- a bias value for the linear ranking mechanism 7: $\tilde{c}$, *p_c_*- crossover operator and crossover rate 8: $\tilde{m}$, *p_m_*- mutation operator and mutation rate 9: **Output** *s_t_*- evolved set of solutions 10: **Begin** 11: *s*_0_ ← *G*enerate*R*andom*S*olutions(*M_k_*) 12: *t* = 0 13: **while** !*SC* **do** 14: *E*valuate(*f*, *s_t_*) 15: *parents_t_* = *S*electParents($\tilde{s}$, *s_t_*, *z*) 16: *C*ompute *DPD* according to the formula (9) 17: *U*pdate *p_c_* according to the formula (10) 18: *children_t_* = *R*ecombination($\tilde{c},$, *p_c_*) 19: *C*onstruct new population $s_{t}^{'}$ from parents and offspring 20: *U*pdate *p_m_* according to the formula (11) 21: *M*utate $s_{t}^{'}(\tilde{m},\;p_{m})$ 22: $s_{t}\leftarrow s_{t}^{'}$ 23: *t* = *t* + 1 24: **end while** 25: **return** *s_t_* 26: **End**

Population will evolve according to the IAGA strategy constantly, until the optimal solution appears. This paper uses two conditions to stop the algorithm:

1. Recording the coverage information of individual traversing test path nodes. When the specific target path is covered, and then find the optimal solution, the end of the algorithm;
2. Because of some test path node may be difficult to cover or can’t cover, you need to set certain evolution algebra, when the number of iterations of the evolution is reached the preset value, the algorithm is end.

## Results and discussion

In this section, the proposed method (IAGA) is applied to generate test case for covering paths of a benchmark and six industrial programs in C. To test the effectiveness of IAGA, traditional method such as random method and other evolutionary generation of test case [10, 16] is taken for comparison. Our computer configuration is Intel(R) Core(TM) i7-6500U Duo CPU @ 2.5GHz, 120GB hard drive and 8GB of DDR4 memory under windows 10 operating system.

### Evaluation criterion and parameter setting

The decision for termination criteria is that if at least one test datum has been found to traverse the target path or the number of iterations of the evolution is reached the preset value, the evolution will stop. Some evaluation criterions to test the effectiveness of different methods are listed as follows:

*Evals*: the number of evaluation for individual evaluation of each method
*T*: the search time for test data generation of each method
*SR (Success Rate)*: the experiments percentage success in generating test datum to traverse the target path for the total number of experiments

In order to ensure that sampling individual differences as small as possible, each method will adopt the same population size and initial population. We will adopt binary coding for individual coding, the linear rank method for selection operator, one point crossover and one point mutation. In the set of genetic parameters, the crossover rate and mutation rate were assigned the values 0.9 (*P*_*c*0_ = 0.9in IAGA) and 0.2 (*P*_*m*0_ = 0.2), respectively. For the proposed method, the weighting parameter ρ for calculating *degree of population diversity DPD* was set 0.5 to make comparison with other approaches. In fact, the weighting parameter ρ can get a good result at a range of [0.4, 0.6] in our experiments.

### Benchmark experiments

The benchmark program is triangle classifier program in Fig 2 which kind of triangles they represent, *i*.*e*. non triangle, scalene, isosceles or equilateral. We choose a difficult path which represents equilateral as our target path, and P(target) = "s → 1,2,3,4,5,6,7,9,10,11,16 → e". We selected the triangle classification program with different input domains and when the input range of the three variables was [1, *N*], the search range reached [1, *N*^3^]. There were *N*^3^ data items in this search space, and the number of data items needed to generate the equilateral triangle was only *N*. Therefore, the probability of generating these scarce data is 1/*N*^2^, which indicated the difficulty of generation with increase in *N*. The experiments setting and results are shown in Tables 2 and 3. To avoid the influence of the randomness of each algorithm, experiments are configured to run 20 times for each group of approach.

**Table 2:** Experiments settings and results of triangle classifier program.

Experiments settings			IAGA			IGA[16]			GA[10]		
Input range	Pop size	Max Gen	Mean *Evals*	Mean *T*(s)	*SR*/%	Mean *Evals*	Mean *T*(s)	*SR*/%	Mean *Evals*	Mean *T*(s)	*SR*/%
[1, 128]	50	5000	6055.3	0.0007	100	51453.0	0.0058	100	102267.8	0.0103	100
[1, 256]	50	10000	9273.5	0.0011	100	132890.3	0.0165	100	270504.0	0.0288	100
[1, 512]	100	20000	25960.0	0.0036	100	512376.0	0.0628	100	1041490.3	0.1443	85
[1,1024]	200	40000	83675.5	0.0121	100	1950530.5	0.3245	90	4018579.5	0.5197	80
[1,2048]	200	60000	161840.2	0.0218	100	4708368.6	0.9142	75	8398830.1	1.6137	60
[1,4096]	200	70000	259080.3	0.0359	100	6151690.4	1.2047	60	13114160	2.5188	25
[1,8192]	200	80000	588118.4	0.0854	100	7110541.9	1.3944	50	15213086	2.9135	15
[1,16384]	300	80000	1104897	0.1643	100	12087919	2.5018	35	22000000	3.2251	0
[1,32768]	300	90000	2120882	0.3153	85	21758254	3.9824	20	24000000	3.6911	0

**Table 3:** Results obtained in the experiment comparison between IAGA and Random approach.

Experiments settings			IAGA			Random		
Input range	Pop size	Max generations	Mean *Evals*	Mean *T*(s)	*SR*/%	Mean *Evals*	Mean *T*(s)	*SR*/%
[1, 128]	50	5000	6055.3	0.0007	100	115249.5	0.0007	100
[1, 256]	50	10000	9273.5	0.0011	100	482368.5	0.0031	100
[1, 512]	100	20000	25960.0	0.0036	100	1506006.0	0.0111	80
[1, 1024]	200	40000	83675.5	0.0121	100	5249142.3	0.0379	55
[1,2048]	200	60000	161840.2	0.0218	100	11014836.7	0.0798	35
[1,4096]	200	70000	259080.3	0.0359	100	20000000.0	0.1432	0
[1,8192]	200	80000	588118.4	0.0854	100	22000000.0	0.1636	0
[1,16384]	300	80000	1104897.0	0.1643	100	22000000.0	0.1636	0
[1,32768]	300	90000	2120882.0	0.3153	85	24000000.0	0.1841	0

**Fig 3:**
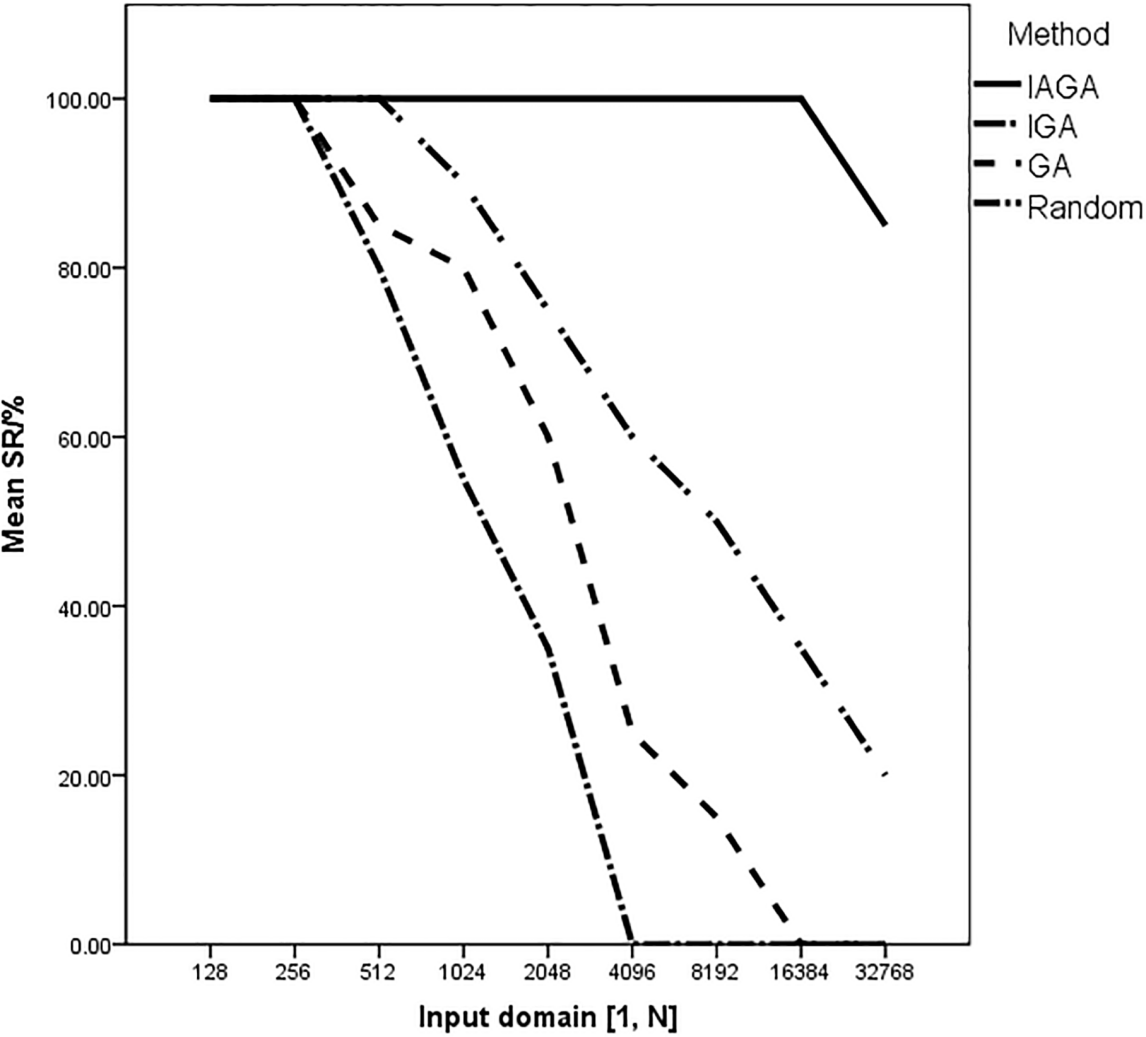
The success rate (SR/%) to generate data of equilateral triangle in different input domain.

We compared the results from Tables 2 and 3 that we can see:

1. The IAGA method this paper proposed has succeed in generating datum to traverse the target path with the fewest evaluations for each input domain. For example, as the input range is [1, 256], the mean evaluations of IAGA is 9273.5 while the mean evaluations of IGA is 132890.3 (about 14.3 times compared to IAGA), the mean evaluations of GA is 270504.0 (about 29.1 times compared to IAGA) and the mean evaluations of the Random approach is 482368.5 (about 52.0 times compared to IAGA).
2. For the search time to each approach, the mean *T*(s) of IAGA is 0.0007 seconds (the same to the Random method) as the input domain is [1, 128], besides that range, the less search time compared to other methods. We can conclude that the random method makes much less time consumption relative to the number of evaluation, this is due to it does not involve the computing time of fitness value for each individual and they are generated by random. The evaluations of IGA and GA are less than the Random method, but it consumes more time for test data generation of each iteration process due to the genetic operators and fitness calculation, and the total search time is more. Although the general process of evolution of IAGA method based on the genetic algorithm similar to IGA and GA, the total number of evaluation greatly reduced due to the adaptive parameters adjustment for evolutionary computing, which makes the total search time *T*(s) least. It shows that the proposed method makes a great improvement in optimization efficiency.
3. As the input domain is a small range, each method (IAGA, IGA, GA and the Random approach) can generate test data to traverse the target path of equilateral triangle. However, the IGA and traditional methods (simple GA and Random approach) have failed in generating test data to cover the target path several times with the growing input range. For example, the *Success Rate* (*SR*) of IGA, GA, and Random method are 90%, 80% and 55% respectively in the input range of [1, 1024]. In fact, the advantages of the IAGA are more obvious with the scale increase of problem. The comparison results in terms of the *Success Rate* (*SR*/%) of searching for scarce data to generate the equilateral triangle in different input domain ranges with the four test methods are shown in Fig 3.

### Industrial programs of experiments

In order to further verify the effectiveness of the proposed method, we choose 6 industrial cases [26] for experiments, and a feasible path is randomly selected as the target path for each industrial program. The description and the corresponding parameter settings are showed in Table 4. LOC is the number of lines of code.

**Table 4:** Description and the corresponding parameter settings to target path of six industrial programs.

Program ID	Program Name	LOC	Nodes of Target Path	Population Size	Max Generations
PG1	space (fixgramp)	90	18	100	2000
PG2	space (fixgrid)	115	12	100	1500
PG3	tot_info	406	53	100	20000
PG4	replace	564	85	200	30000
PG5	sed	8063	382	200	50000
PG6	flex	11783	543	400	50000

In this group of experiments, each method will run 50 times independently. The experimental statistical results of evaluations are shown in Table 5, and the results of the mean search time and success rate (*SR*/%) are shown in Table 6.

**Table 5:** Experiments results of evaluations of six industrial programs.

Program ID	Evaluations of Different Methods							
	IAGA		IGA		GA		Random	
	Mean	Standard Deviation	Mean	Standard Deviation	Mean	Standard Deviation	Mean	Standard Deviation
PG1	7539.1	3038.4	8946.5	5103.2	12081.8	6911.2	14454.8	9455.2
PG2	5223.1	2911.7	7119.8	4885.6	11604.7	5648.5	13596.4	8322.7
PG3	414279.3	13256.4	532456.5	27833.6	627919.2	19465.0	954291.6	32445.6
PG4	479219.4	16845.6	877419.7	19946.4	1074568.1	26572.2	1396923.7	57701.6
PG5	712858.5	245741.3	1036656.6	204674.2	2249018.2	498529.6	4414326.5	1089441.4
PG6	3350432.1	540630.2	4353955.2	594898.6	7421759.4	697940.6	8711123.2	4521180.1

**Table 6:** Experiments results of mean search time and success rate of six industrial programs.

Program ID	IAGA		IGA		GA		Random	
	Mean *T*(s)	*SR*/%	Mean *T*(s)	*SR*/%	Mean *T*(s)	*SR*/%	Mean *T*(s)	*SR*/%
PG1	0.0254	100	0.0278	100	0.0320	100	0.0330	100
PG2	0.0265	100	0.0310	100	0.0339	100	0.0341	100
PG3	7.0819	100	7.0744	100	7.8142	76	4.7795	42
PG4	21.2090	100	24.3842	92	33.5051	64	8.7241	26
PG5	41.8687	96	57.1118	84	53.0032	48	12.4133	16
PG6	61.0240	90	72.3125	78	80.6806	38	23.1058	18

**Fig 4:**
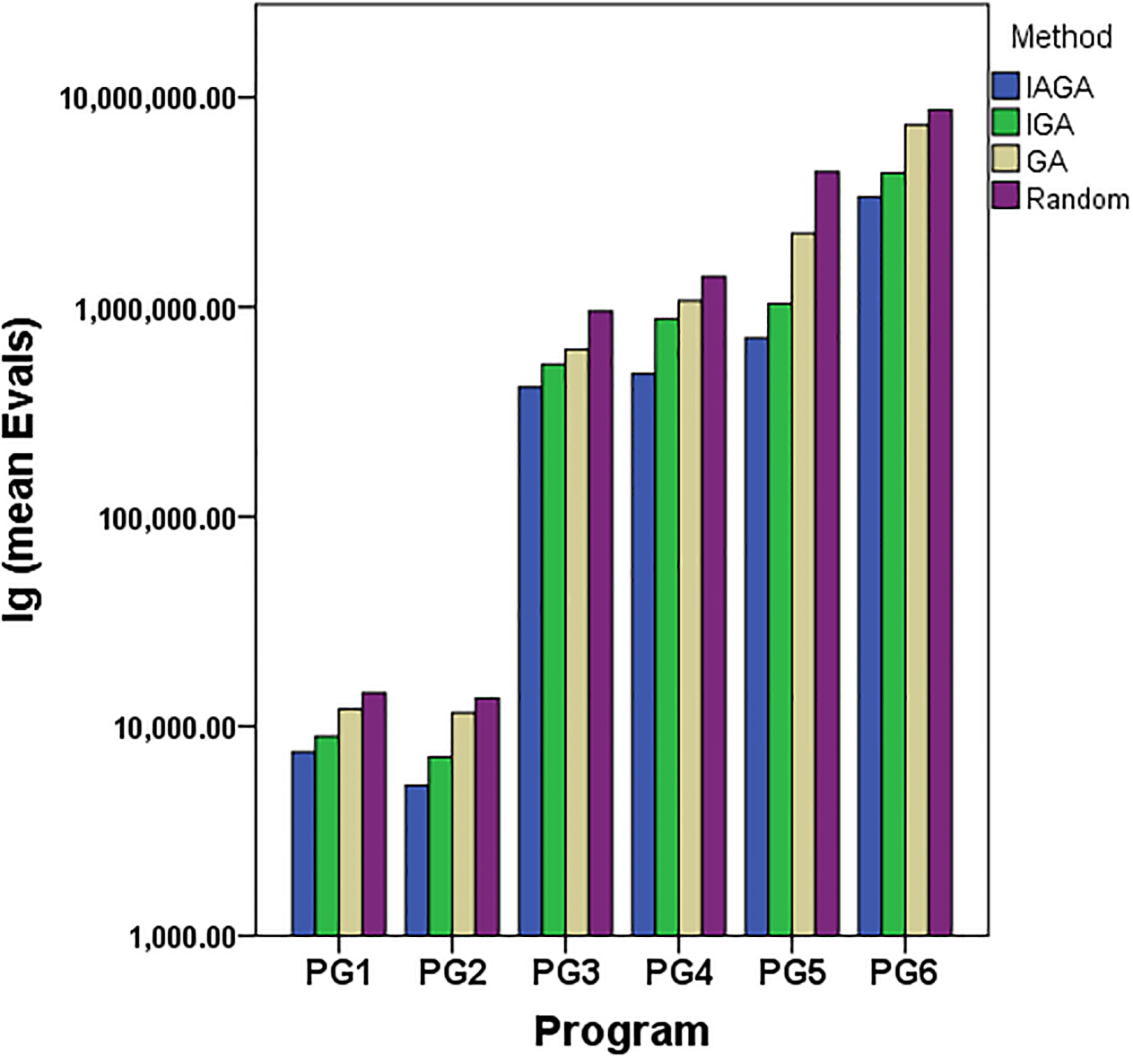
The lg(mean Evals) of six industrial programs with four methods.

We compared the results from Table 5 that we can see:

1. In terms of the mean evaluations, the proposed method for generating path-oriented data (IAGA) is still less than other methods (IGA, GA, and the Random approach) for the six industrial programs. The Fig 4 is depicted to compare the difference of the *Evals* with each method clearly, which the ordinate is the logs base 10 of the mean evaluations because the sizes of these programs are intervallic. As we can see in Fig 4, with the increase scale of program, the advantage of evaluations for IAGA is more obvious.
2. In terms of the standard deviation of evaluations of the six industrial programs, the IAGA method is the least compared to other three methods. And the standard deviation of evaluations with IGA is less than GA, while the Random method is the most for each program under test. It indicates the performance of stability for IAGA is better than other three methods, and the randomness of the Random method is obvious with the scale of problem increasing.

We can see from Table 6 that the results of the search time *T*(s) of the six industrial programs for IAGA is not always better than other three methods. In fact, for PG3~PG6, the Random method can get the greatest ‘grades’ in terms of the mean search time. However, for the ‘space’ programs PG1 and PG2 in which each of these four methods can generate test data to traverse the specific target paths successfully, the mean search time of the IAGA method is the best. When compared to other two evolutionary algorithms, the mean search time of the proposed method is less than the IGA and GA method for the six industrial programs except the PG3 (‘tot_info’ program). For the ‘tot_info’ program, the IAGA method takes 7.0819 seconds and it is slightly higher than the IGA method which takes 7.0744 seconds.

**Fig 5:**
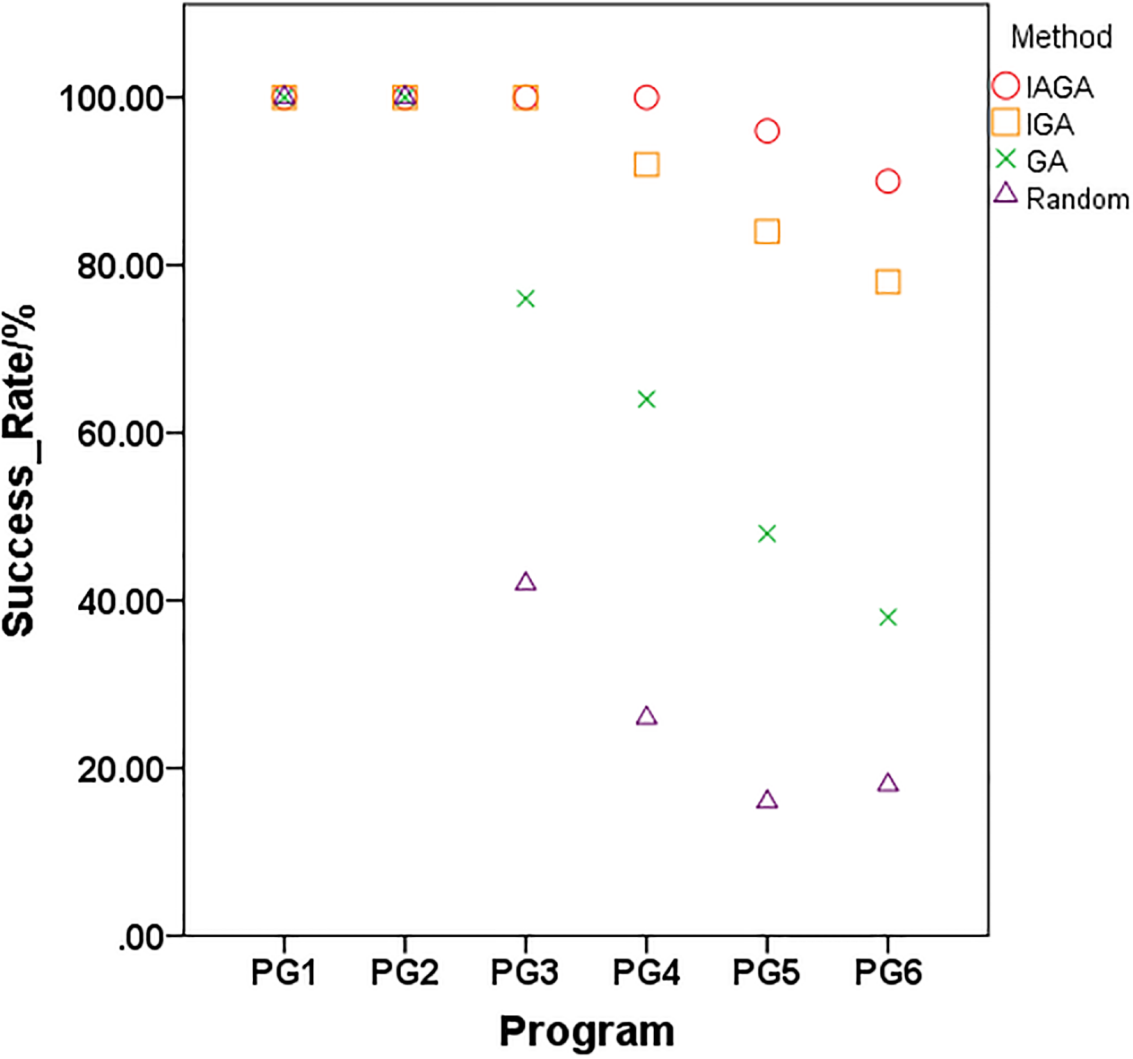
The success rate of six industrial programs with four methods.

The success rate comparison results of the six tested programs with four methods as shown in Fig 5 from Table 6.

In terms of the success rate, for PG1 and PG2, each of these four methods achieve test data generation to traverse the target path (all the *SR*/% are 100%) because the two functions of the ‘space’ program are relatively simple. For the complex programs ‘tot_info’ and ‘replace’ with more target path nodes, the difficulty of the test data generation is higher. The success rate of the GA method and Random method are 76% and 42% respectively for PG3, and the success rate of the IGA method, GA method and Random method are 92%, 64% and 26% respectively for PG4, while the proposed method (IAGA) is still 100% for them. For the large-scale programs PG5 and PG6 (‘sed’ and ‘flex’), although these four methods have failed in generating test data for 50 trials, the success rate of the IAGA method and IGA method are much higher than the GA method and Random method, and the proposed method is the best of them.

## Conclusions

This paper proposed an automatic test case generation method based on an improved genetic algorithm. It improves search efficiency by maintaining population diversity according to adjusting crossover rate and mutation rate in a dynamic way. The experimental results show that the proposed method (IAGA) is more effective than existing similar methods and random method for path testing. Although the subjects selected in this paper are C language, the thought of this method can be used for reference in other language as experimental objects. Further study in this area should include discussions of designing adaptive genetic operators and searching for an appropriate fitness function when considering defect detection.

## Supporting information

S1 Data The initial data of Fig 3. The success rate (SR/%) to generate data of equilateral triangle in different input domain.
(XLSX)

S2 Data The initial data of Fig 4. The lg(mean Evals) of six industrial programs with four methods.
(XLSX)

S3 Data The initial data of Fig 5. The success rate of six industrial programs with four methods.
(XLSX)
